# Palm-Plant Pain, Sign of a Severe Systemic Disease? Case Report and Review of Literature

**DOI:** 10.3390/genes14020516

**Published:** 2023-02-17

**Authors:** Iuliana Magdalena Starcea, Lavinia Bodescu Amancei Ionescu, Tudor Ilie Lazaruc, Vasile Valeriu Lupu, Roxana Alexandra Bogos, Ileana Ioniuc, Felicia Dragan, Ancuta Lupu, Laura Stefana Galatanu, Ingrith Crenguta Miron, Adriana Mocanu

**Affiliations:** 1 Pediatrics, “Grigore T. Popa” University of Medicine and Pharmacy, 700115 Iasi, Romania; 2 Faculty of Medicine and Pharmacy, University of Oradea, 410087 Oradea, Romania

**Keywords:** palm-plant pain, end stage renal disease, Fabry disease, children

## Abstract

Fabry disease is an X-linked lysosomal storage disease, second in prevalence after Gaucher disease. The onset of symptoms occurs in childhood or adolescence with palmo-plantar burning pains, hypo hidrosis, angiokeratomas, and corneal deposits. In the absence of diagnosis and treatment, the disease will progress to the late phase, characterized by progressive cardiac, cerebral and renal damage, and possible death. We present the case of an 11-year-old male boy who was transferred to the Pediatric Nephrology Department for palmo-plantar burning pain and end stage renal disease. Following the evaluations for the etiology of end stage renal disease we excluded the vasculitis, the neurologic diseases, extrapulmonary tuberculosis. Because of suggestive aspect at CT scan and lack of etiologic diagnosis of renal insufficiency we performed lymph node and kidney biopsy, with a surprising result for storage disease. The specific investigation confirmed the diagnosis.

## 1. Introduction

Fabry disease is an X-linked lysosomal storage disease, second in prevalence after Gaucher disease. It does not take into account ethnicity or race, with an average incidence of approximately 1:117,000 births [1]. Recent studies carried out through neonatal screening have demonstrated a higher prevalence, from 1:3000 to 1:7800 male newborns (even 1:1500 in Taiwan) [1,2,3]. The mutation of the gene that codes for the synthesis of α—galactosidase A leads to enzyme deficiency and generates systemic deposits of glycosphingolipids that cause neurological, renal, cardiac and cerebrovascular dysfunctions [1]. The disease manifests itself in hemizygous boys or heterozygous girls. The onset of symptoms occurs in childhood or adolescence with palmo-plantar burning pains, hypo hidrosis, angiokeratomas, and corneal deposits. In the absence of diagnosis and treatment, the disease will progress to the late phase, characterized by progressive cardiac, cerebral, and renal damage leading to multiple organ dysfunction and death [1,4,5].

## 2. Case Presentation

We present the case of an 11-year-old male boy who was transferred to the Pediatric Nephrology Department for burning pain in the palms and plants, marked asthenia, selective anorexia for meat, eyelid edema, and vertigo.

The medical history attests the presence in the last 2 years of moderate normocytic normochromic anemia (Hb 8 g/dL), associated with proteinuria and microscopic hematuria. He also had repeated episodes of acute angina. From the family history, we recall both parents diagnosed with tuberculosis 4 years ago, which is why the patient underwent a prophylactic treatment with isoniazid for 6 months.

The patient was investigated in a regional hospital, where facial edema, high blood pressure 155/90 mmHg, severe anemia (Hb 7.2 g/dL), market nitrogen retention (urea 376 mg/dL, creatinine 14.7 mg/dL, GFR 4.71 mL/min/1.73 m), proteinuria, and microscopic hematuria in the urine summary were prominent.

Anthropometric data at the presentation in our clinic showed a marked height-weight hypotrophy, −2.5 standard deviations. The patient had a poor general condition, significant palmo-plantar pain with a burning character, pale skin and mucous membranes, and palpebral and pretibial edema. It also associated high blood pressure (140/90 mmHg, +10 mmHg up to the 97.5th percentile for the waist), with systolic murmur III/6 at the apex, as well as oliguria—200 mL urine/24 h.

Biological investigations revealed the presence of an important nitrogen retention (urea 270 mg/dL, creatinine 16 mg/dL and a GFR of 4 mL/min/1.73 m²) associated with severe hypo regenerative normochromic normocytic anemia (Hb 6.8 g/dL, Ht 20.5%, MCV 80.8 µ³, MHC 29.7 pg%, MCHC 36.8%, Reticulocytes 20‰), with high serum iron 125γ%, and ferritin 611 ng/mL, metabolic acidosis (13 mmol/L), hypocalcemia (0.4 mmol/L), hyperphosphatemia (6.64 g/L) and high alkaline phosphatase—1897 IU/L. These changes attested an advanced renal failure, complicated with renal osteodystrophy and severe anemia. We tried to correct with erythrocyte mass transfusion, and after that initiated erythropoietin treatment for secondary renal anemia. There were no changes in the liver tests, proteinemia and lipid profile. He associated nephritic range proteinuria (1.2 g/24 h).

**Figure 1:**
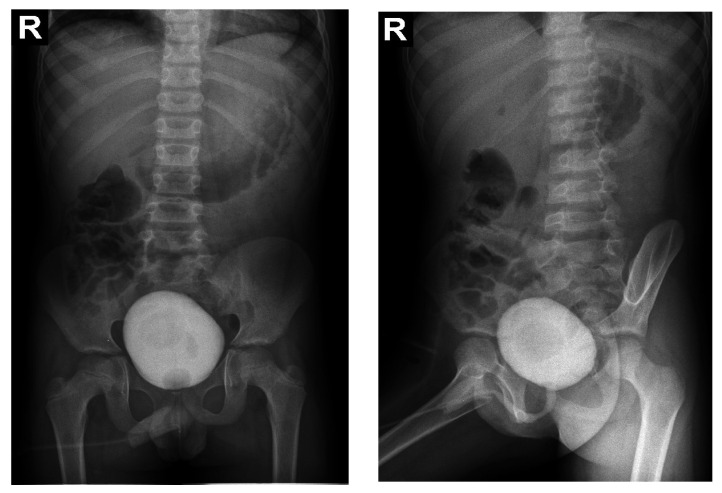
Voiding mictional cystourethrogram.

Due to the poor medical condition, with advanced renal failure, acidosis, arterial hypertension, the patient required the urgent initiation of hemodialysis. At the same time, we continued investigations for the etiology of renal failure (Table 1). We excluded the reflux nephropathy (secondary to primary or secondary vesicoureteral reflux, or posterior urethral valve) by voiding cystourethrogram (Figure 1).

**Table 1:** Imaging tests.

Investigations	Result
Abdominal ultrasound	▪ bilateral renal hypotrophy, RK 6.5/2.1 cm, LK 7.1/2.56 cm (normal size at 11 years 10 cm); multiple homogenous hyperechoic nodular formations; at the lower pole of the left kidney a solid, homogeneous nodular formation with a diameter of 2.9/2.8 cm
Abdominal-pelvic computed tomography with contrast substance (Figure 2)	▪ delayed and symmetrically reduced renal secretion ▪ are described expansive renal formations replacing renal parenchyma and with a mass effect on it
Kidney biopsy	▪ Chronic glomerulonephritis with segmental and diffuse glomerular hyalinization (sclerosis) (Figure 3) ▪ Rare glomeruli with fibrinoid deposits in the mesangium and outstanding endothelial cells with a swollen appearance are observed (Figure 4) ▪ Renal tubes have vacuolated cytoplasm (possible in the context of lipid storage), negative PAS. There are rare foci of chronic inflammation around some tubes (Figure 5) ▪ Arteriole-type vessels have thickened walls due to swelling of the endothelium and vacuolization of muscle cells. Arterioles with fibrinoid deposits also appear (Figure 6) ▪ IF: IgA and IgM present in massive glomerular deposits and in the hyaline cylinders in the tubules. C3, kappa, lambda and Fg present in glomeruli
Chest X-ray (Figure 7)	▪ accentuated pulmonary pattern and a heart with an increased transverse diameter
Electrocardiography ECG (Figure 8)	▪ sinus rhythm 75/min ▪ QRS axis at + 30° ▪ QRS = 0.10 s ▪ subendocardial myocardial ischemia
Echocardiography (Figure 9)	▪ mitral insufficiency second degree ▪ aortic insufficiency first degree ▪ tricuspid insufficiency first degree ▪ dilatation of the left heart ▪ ejection fraction 50% ▪ fine pericardial reaction, 3 mm
Voiding mictional cystourethrogram (Figure 1)	▪ excluded the presence of a posterior urethral valve or primary vesicoureteral reflux
IDR test (tuberculin intradermal reaction)	▪ value of 22 mm Palmer III—suggests tuberculin turn
Uroculture for Koch bacillus	▪ negative
Gastric lavage for Koch’s bacillus	▪ negative

**Figure 2:**
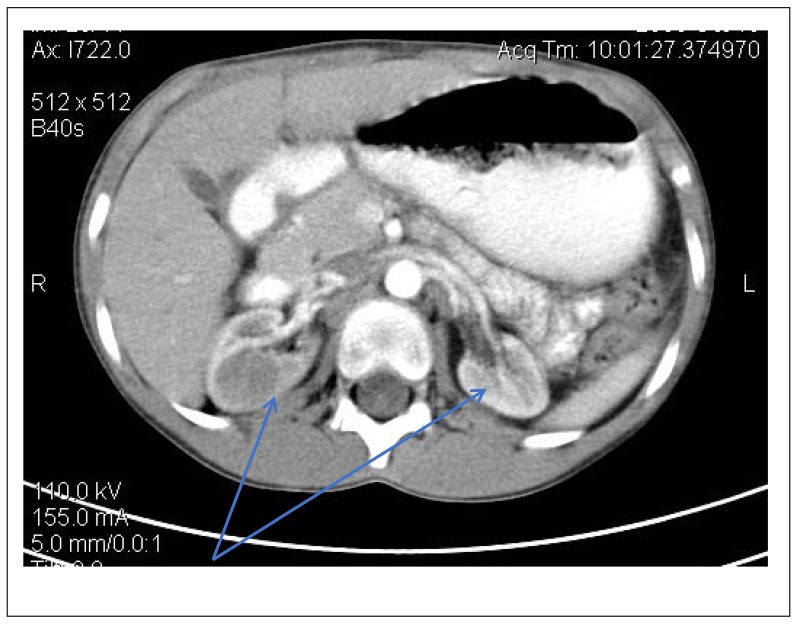
Abdominal CT scan—expansive renal formations replacing renal parenchyma and with a mass effect (blue arrow).

**Figure 3:**
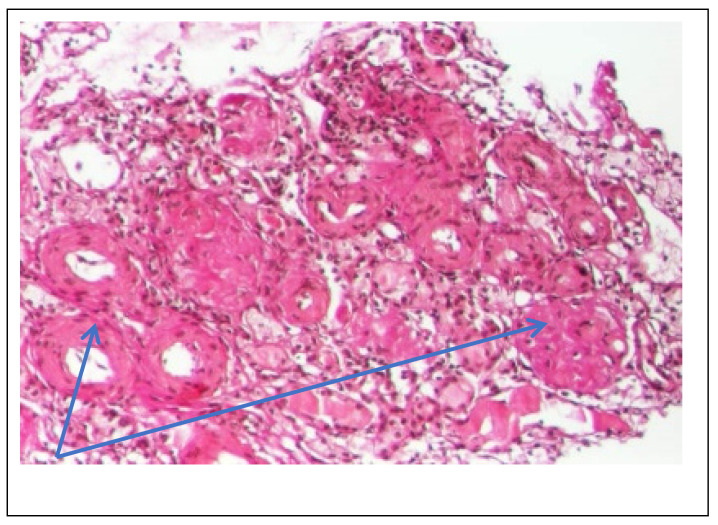
Kidney biopsy (*hematoxylin*-*eosin* staining × 100)—Glomeruli and hyalinized vessels (blue arrow).

**Figure 4:**
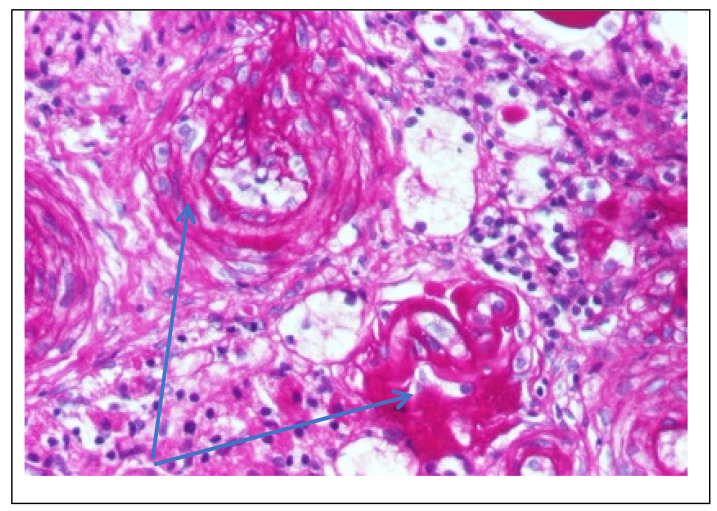
Kidney biopsy (*PAS* staining × 200)—Fibrinoid deposits in the glomerulus and PAS+ wall thickened vessel (blue arrow).

**Figure 5:**
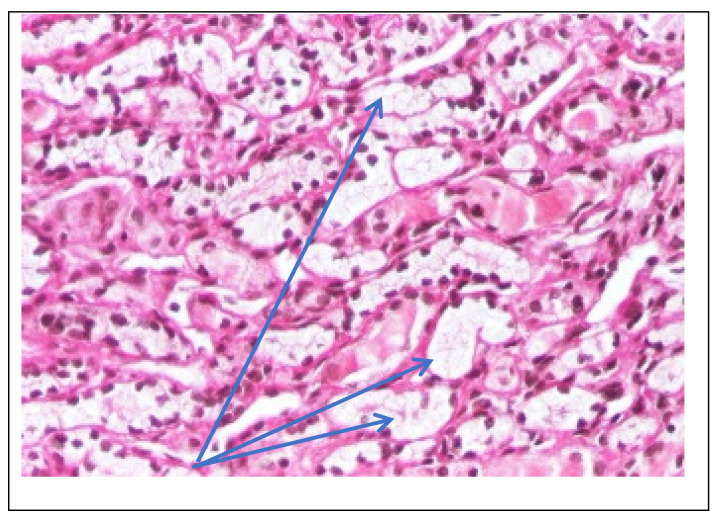
Kidney biopsy (*hematoxylin*-*eosin* staining × 200)—Distal tubes with intracytoplasmic vacuoles (blue arrow).

**Figure 6:**
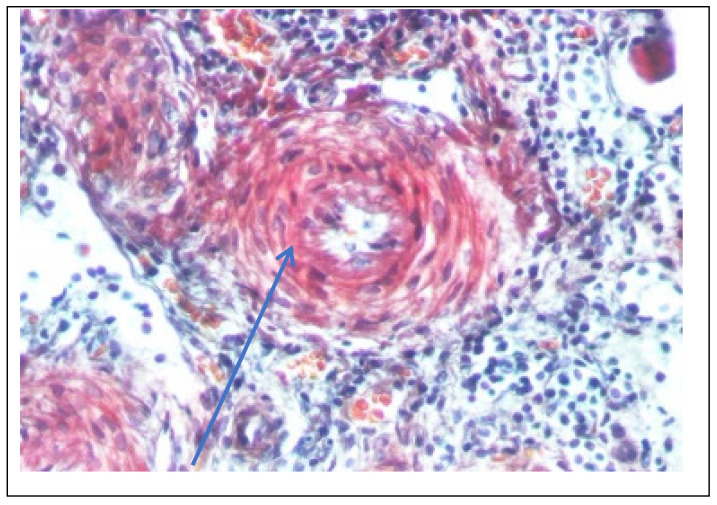
Kidney biopsy (Trichrome staining × 200)—Vessel with thickened wall (blue arrow).

**Figure 7:**
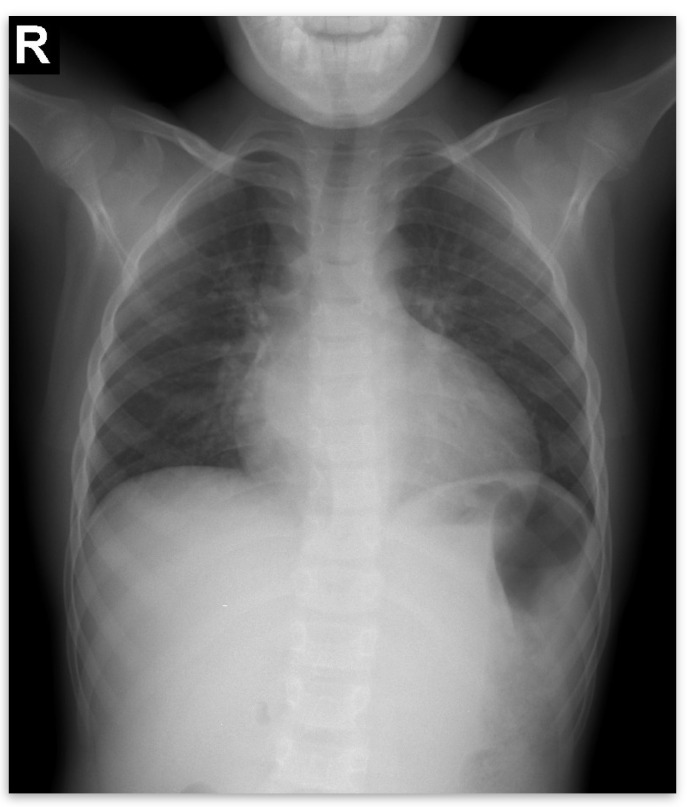
Chest X-ray.

**Figure 8:**
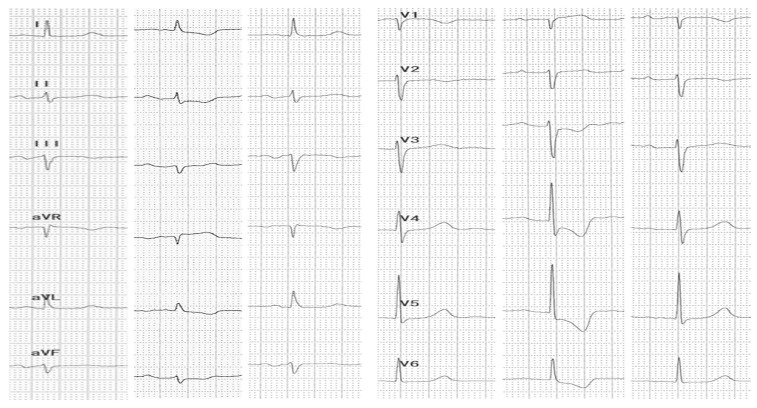
ECG—ST-segment depressions and T-wave inversions.

**Figure 9:**
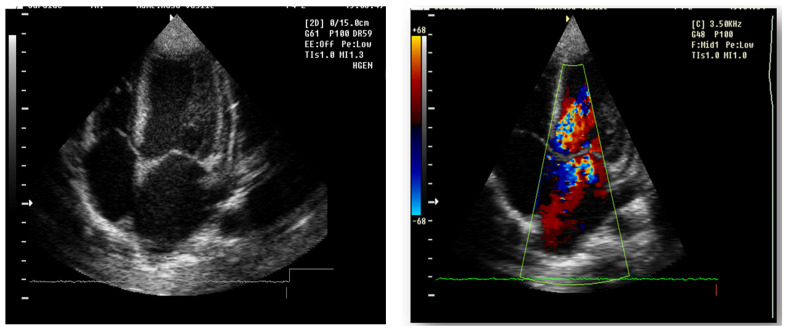
Echocardiography 4 chamber transthoracic apical view: echodense mitral and aortic valve, II-degree mitral regurgitation and I degree aortic regurgitation, pulmonary and tricuspid valve echodense, with I degree regurgitation. Left atrium and Left ventricle dilatation, normal kinetics, EF 70%, inferior vena cave not dilated, with inspiratory collapse, E/A > 2.

Following the evaluations for the etiology of end stage renal disease (ESRD) we recommended abdominal ultrasound and CT scan. The abdominal ultrasound showed multiple homogenous hyperechoic nodular formations. The CT scan showed expansive renal formations replacing renal parenchyma, with a mass effect on it (Figure 2). We thought in that moment of a bilateral nephroblastoma, but the clinical and biological dates excluded it. There remained a possibility of extrapulmonary renal tuberculosis (due to his personal and family history). Even if the culture was negative for Koch Bacillus, while a value of 22 mm Palmer III—suggests tuberculin turn, the treatment was initiated according to the international guidelines [6] by the phthisiologist: triple combination of isoniazid, rifampicin and pyrazinamide in doses adjusted to his clearance, in the 7/7 scheme for 2 months, then isoniazid and rifampicin in the same doses 7/7 for another 7 months [6]. Two months after the initiation of the tuberculostatic treatment, a CT reevaluation was performed, which showed no changes in the renal formations, although biologically the inflammatory syndrome was absent. The child continued the chronic dialysis program and still had important palm-plant pain burning type. The suspicion of chronic glomerulonephritis with evolution towards end-stage renal failure remained, so, we decided to perform the kidney biopsy who showed Chronic glomerulonephritis with segmental and diffuse glomerular hyalinization (Table 1, Figure 3, Figure 4, Figure 5 and Figure 6).

**Figure 10:**
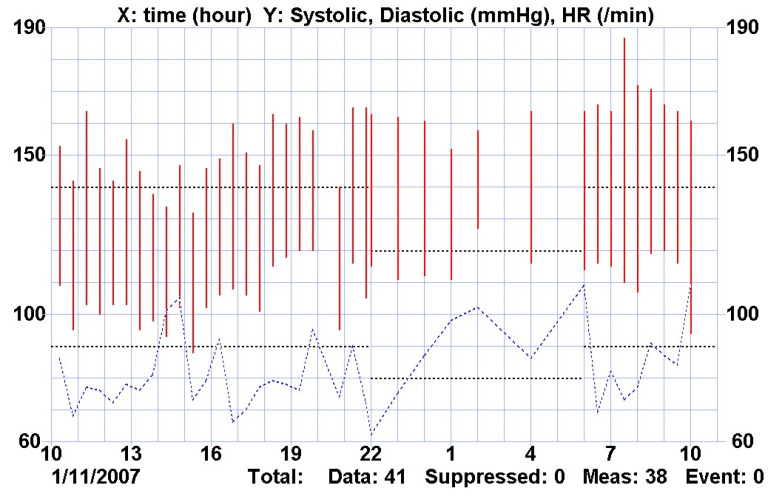
Holter BP.

In evolution, the patient’s hypertension acquired a permanent character, being observed both in manual measurements and in continuous recordings (Figure 10), with arterial hypertension values of 150–170/100–120 mmHg. It was necessary to start antihypertensive treatment in a quadruple combination: angiotensin-converting enzyme inhibitor (Enalapril) + selective calcium channel blocker (Amlodipine) + selective β blocker for β_1_ receptors (Metoprolol) + α_1_ blocker (Prazosin), simultaneously with weight reduction by dialysis to ideal weight preset by Body Composition Monitoring (BCM).

Forty-six days after admission, the patient suddenly complained one morning of epigastric pains, nausea and palmo-plantar burning, associated with a hypertensive episode (BP 180/110 mmHg), for which reason a hemodialysis session was performed urgently, extracurricular. In the same evening, at a BP value of 160/100 mmHg, at rest, the patient presented a sudden and severe worsening of the general condition, dyspnea with orthopnea, intense wheezing, SaO_2_ 89–91%, tachycardia 140/min, subcrepitants disseminated on both lung areas. The suspicion of cardiogenic acute pulmonary edema was confirmed by imaging (chest X-ray—Figure 7, Table 1). Electrocardiographic evaluation (Figure 8, Table 1) revealed subendocardial myocardial ischemia, and echocardiography revealed acute dilation of the left heart, as well as a fine pericardial reaction, 3 mm, without signs of cardiac tamponade (Figure 9, Table 1). At that moment, we made 2-h ultrafiltration, during which the signs of acute pulmonary edema subsided. After 60 min an increase in blood pressure up to 160/120 mmHg is noted, with the maintenance of negative T waves on the monitor. Hemodialysis was stopped and iv Nicardipin was administered, with good results.

The patient repeated in the following days lower precordial and retrosternal pain with radiation in the epigastrium, accompanied by nausea and vomiting and palmo-plantar burning, associating ECG changes of subendocardial ischemia, not significantly influenced by the sublingual nitroglycerin administration, especially during extrarenal hemodialysis. Repeated determinations of creatine kinase (CK), CK-MB, Troponin I, Alanine aminotransferase, Glutamate oxaloacetate transaminase, LDH were normal and ruled out an acute coronary syndrome.

Considering the deterioration of cardiac function with the maintenance of changes on electrocardiography and the lack of a favorable response to treatment, it was decided to reanalyze the case and the following possible diagnoses were raised: periarteritis nodosa, collagenosis, secondary amyloidosis, storage disease or light-chain deposition disease.

Serological tests (Table 2) were negative.

At the same time the child needed to be converted to peritoneal dialysis, because all the cardiac manifestations developed in the hemodialysis sessions.

**Figure 11:**
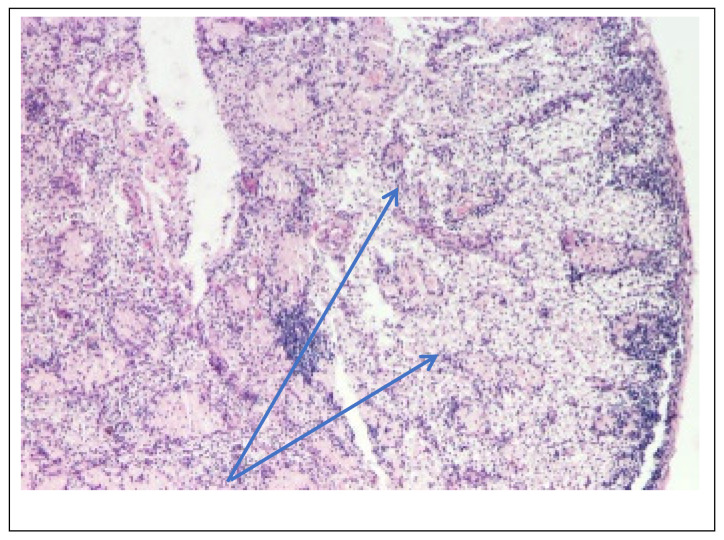
Mesenteric ganglion biopsy (*PAS* staining × 100)—Lymph node with obliterated architecture (blue arrow).

**Figure 12:**
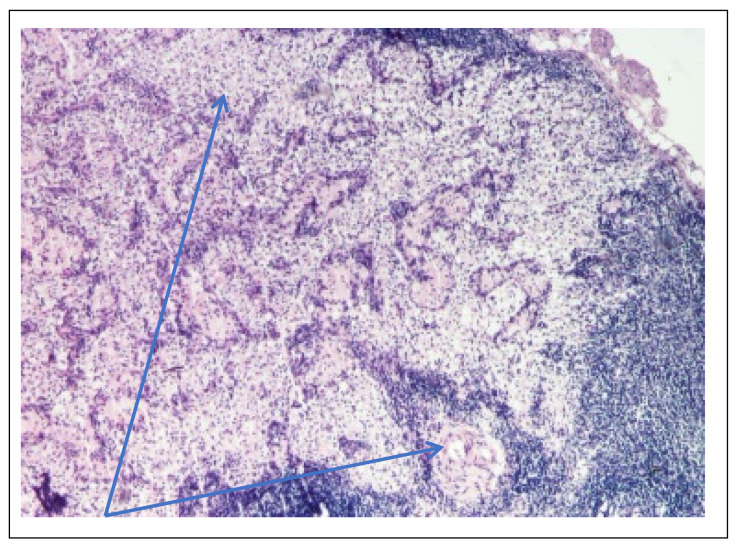
Mesenteric ganglion biopsy (*PAS* staining × 100)—areas of macrophages with foamy cytoplasm (blue arrow).

On the occasion of the placement of the peritoneal dialysis catheter, a lymph node was sampled, which was later analyzed by optical microscopy, PAS staining and hematoxylin eosin staining. The result showed complete deletion of the normal architecture of the ganglion (Figure 11, Table 3), nests of lymphocytes and remnants of lymphoid follicles, areas of macrophages with foamy cytoplasm (Figure 12, Table 3), beaches of macrophages with eosinophilic, homogeneous cytoplasm, like in storage disease.

Analysis of biopsy samples (Table 3) and ophthalmological examination (Table 3) were performed, with the outline of a possible storage disease, which is why we tested the enzymes responsible for neurolipidosis (Gaucher disease, Schindler, Fabry, Landing/Morquio B, Sandhoff, Tay Sachs, metachromatic leukodystrophy, multiple sulfatase deficiency), mucopolysaccharidoses, glycoproteinoses (α fucosidosis, α mannosidase, β mannosidase), and mucolipidoses.

After recording a significant reduction in serum α-galactosidase activity 0.25 nmol/h/mL (N: 7–20 nmol/h/mL plasma) and leukocyte α-galactosidase activity 1.68 nmol/h/mg proteins (N: 100–800 nmol /h/mg proteins) the positive diagnosis of Fabry disease is established, which includes renal (ESRD), cardiac (valvular and myocardial damage, left heart hypertrophy), neurological (acroparesthesia) and ocular (vortex keratopathy—Table 3) damage.

Genetic testing GLA variants revealed c.317T>G mutation, variant p.Leu106Arg, and the molecular consequence was Missense. The same mutation was detected in his mother also.

**Figure 13:**
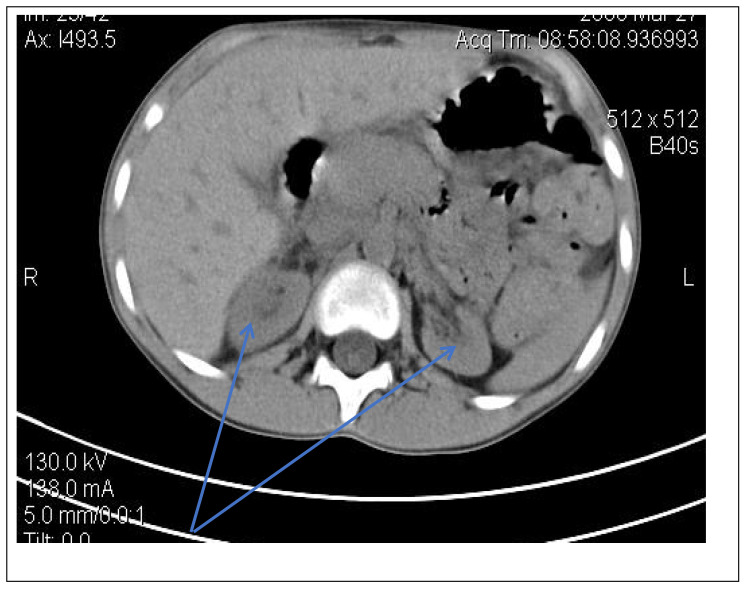
Abdominal CT scan—disappearance of renal hyperechoic formations (blue arrow).

Enzyme replacement therapy (ERT) (Agalsidase β) was subsequently initiated, under which the patient showed a favorable evolution, with the disappearance of palmo-plantar burning acroparesthesias after 3 months of therapy, the normalization of the echocardiographic appearance and the disappearance of left ventricular hypertrophy after 6 months and the disappearance of renal hyperechoic formations on CT-scan after 12 months (Figure 13). Enzyme activity was also tested on the mother and the sister, the mother showing α-galactosidase deficiency.

In evolution, the patient benefited from a kidney transplant after 2 years of renal substitution therapy.

## 3. Discussion

Fabry disease was independently reported by Johannes Fabry and William Anderson in 1898. Fabry disease is most frequently diagnosed in adulthood. This is one of the reasons why the diagnosis is often made late in children. Another reason would be the non-specific symptomatology present at the beginning: acroparesthesias, abdominal pain, changes in intestinal transit or urinalysis (presence of proteinuria or microscopic hematuria). Therefore, in the absence of a positive family history, these changes are not associated with Fabry disease at the beginning [1,7].

The delay in diagnosis affects the patient both on a psychosocial level (leads to marked anxiety) and on a medical level (leads to the appearance of irreversible changes). This also includes the case presented above, where, although the patient had disease-related changes (palmo-plantar acroparesthesias, proteinuria and microscopic hematuria) for about three years, the diagnosis was only considered at the time of the association of progressive cardiac damage.

Severe neuropathic pain is a hallmark of Fabry disease. Patients with Fabry disease experience acute and chronic pain commonly in their hands, feet, and abdomen. The pain experienced by these patients significantly affects their quality of life and their ability to perform everyday tasks. Patients with Fabry disease suffer from acute pain attacks, sensory abnormalities, peripheral neuropathy, or continuous pain throughout life. Although there is pain therapy depending on its scale and enzyme replacement therapy, the pain persists in many of these patients even after starting the specific therapy. Although progress has been made in recent years in understanding the pathogenesis of Fabry disease pain, there is still no consensus on the pain and sensory abnormalities in these patients, nor on the most recommended therapy. Our knowledge is limited in part due to the lack of adequate preclinical models to study the disease. In a recent review, Burand et al. reviewed with the aim of providing an overview of pain in Fabry disease and how preclinical models reproduce aspects of pain seen in patients to better aid future studies of mechanical pain as well as therapy development [8]. Accumulation of globotriaosylceramides in neurons is one of the most important pathological ways to peripheral neuropathy in Fabry disease. The peripheral neuropathy affects over a quarter of patients with Fabry disease and is characterized by loss of small myelinated and nonmyelinated fibers, whereas larger fibers are largely unaffected [8,9]. This loss of fibers is most prominent in the long distal axons of the lower extremities but can also be found proximally in the thigh. Interestingly, studies suggest that fiber loss is substantially greater in the skin than in the peripheral nerve trunk. Previous studies in patients with diabetic peripheral neuropathy have shown a good correlation between intraepidermal nerve fiber density and neuropathic pain intensity [10]. Lower nerve fiber density correlates with greater pain. This negative correlation between intraepidermal nerve fiber density and pain intensity is also observed in patients with chemotherapy-induced neuropathy. However, in Fabry disease, it is not yet clear whether the lower density of intraepidermal nerve fibers is associated with increased pain intensity [9,11].

Considering that pain is an important clinical feature in Fabry disease, clinicians should thoroughly evaluate this manifestation as an integrating part of peripheral nervous system assessment, that should also include somatosensory evaluation, such as quantitative sensory testing, nociceptive evoked potentials and analysis of skin biopsies. Pain should be evaluated with regard to localization (primarily reported in fingers, palms and soles), character and its temporal course, while a quantification can generally be obtained using particular tools, such as Fabry-specific Pediatric Health and Pain Questionnaire (children/adolescents), FabryScan or Wurzburg Fabry Pain Questionnaire (adults) [12]. Among multiple pathophysiological mechanisms generating this symptom, deposits of GL-3 in the dorsal root ganglion neurons are one of the most important, as pain in patients with Fabry disease usually manifests as neuropathic [12]. However, nerve fiber conduction is also modified, mainly implying functional deficits in small unmyelinated fibers [8]. The complexity of these mechanisms give birth to a large spectrum of clinical manifestations, as pain can manifest itself as inflammatory pain, evoked pain, chronic pain or pain crises. The latter install spontaneously in the extremities, frequently as burning pain spreading proximally, being triggered by exercise, fever, exhaustion or changes in ambient temperature [8]. From a somatosensory point of view, taking in consideration the associating sweating disfunction (hypophoresis), an impairment in patient sensation of warmth and cold is another clinical manifestation which might indicate Fabry disease. An important aspect from a pediatrician’s point of view is that in childhood, pain might begin as gastrointestinal in nature, entailing failure to thrive [8] with gastro-intestinal dysmotility possibly installing later in the process.

Not only the pain itself has an impact on the patient with Fabry disease still undiagnosed. The consequences of chronic pain affect the patient’s mental balance, and the most common manifestation is depression. A study conducted in Brasilia on patients with Fabry disease showed that the rate of depression correlates with the intensity of pain [13]. Other studies found that up to 16% of Fabry patients were taking antidepressant medication, more than the general US population [14]. As the intensity of pain increases, it progressively interferes with mood and general enjoyment of life. In our case, the patient underwent depressive-type changes, in the context where the pain was quasi-continuous, long-lasting, significantly impacting the quality of life. Moreover, the child and the family requested hospitalization in the context in which the burning palmar-plantar pain could not be controlled at the level of primary medicine. Later, the impact of the diagnosis of chronic kidney disease in the dialysis stage significantly contributed to the deepening of depression in this case. Depression is generally accepted as the most common psychological problem in chronic kidney disease. Although depressive symptomatology is common in dialysis patients, clinical depression syndrome includes sadness, guilt, hopelessness, helplessness, and changes in sleep, appetite, impacting somatic development. Somatic factors such as uremic toxicity, atherosclerosis, neurological disorders, anemia, cardiovascular disorders, and metabolic disorders are also implicated in the etiology of depression [15]. In general, patients with Fabry disease report that their pain directly and severely affects their quality of life.

Hearing loss, vertigo, cerebrovascular, ocular and dermatologic involvement are also to be noted [16]. Ophthalmologic manifestations can install as soon as the first decade of life and consists mainly of inferior corneal deposits, with linear pigmentation creating a specific finding named cornea verticillate. In addition, Fabry posterior cataract might also install [17]. The verticillate corneal appearance (vortex or spiral keratopathy) supports the diagnosis of Fabry disease and is widely considered a hallmark of the classic form of the disease [18]. After raising the suspicion of storage disease, we performed an ophthalmological examination in the case of our patient, which revealed “Swirling” subepithelial opacities in the lower half of the cornea, highly suggestive of keratopathy associated with Fabry disease.

The major sign of cardiac involvement in Fabry disease is left ventricular hypertrophy, but conduction disturbances such as short PR interval (due to accelerated conduction in the absence of the accessory pathway) and rhythm disturbances such as sinus bradycardia have also been reported. Cardiac ultrasound and ECG are indicated at baseline and at all follow-up visits. Holter testing is recommended only if indicated by symptoms, as severe arrhythmias are not usually encountered in early childhood [18]. In our case, the boy presented dilatation of the left cavities with secondary mitral and aortic regurgitation from the beginning. This could not be attributed only to hypertension secondary to chronic kidney disease, because it regressed after the initiation of enzyme replacement therapy. During the follow-up, he did not present any rhythm or conduction disorders, but the phenomena of myocardial ischemia were observed in the context of hemodialysis, requiring a change in the method of renal replacement.

Regarding dermatologic abnormalities, angiokeratoma is the most common manifestation followed by telangiectasia [19]. The presented boy only had palmo-plantar pain, without the angiokeratomas considered pathognomonic, which caused the diagnosis to be delayed until presentation to our service.

Children with Fabry disease generally do not develop chronic kidney disease until adulthood, when renal failure accounts for much of the morbidity and mortality associated with this disease, particularly in males. Globotriaosylceramide accumulation in renal cells and effacement of podocyte processes can be seen in renal biopsies in children with Fabry disease, even before proteinuria manifests as an early sign of renal involvement [18,20]. In this situation the kidney biopsy with electron microscopy is mandatory to make a differential diagnosis between minimal change nephrotic syndrome and podocyte involvement in context of Fabry disease. In nephrotic syndrome found in electron microscopy the retraction of podocyte processes inside the cell body, with the appearance of a flat epithelial layer [21]. Renal biopsy has been proposed and shown to be safe by several authors and should be considered in selected pediatric cases, especially when the decision to start renal replacement therapy is questionable or in children with significant proteinuria where renal biopsy is essential to rule out a second kidney disease, such as in our case. Focal segmental glomerulosclerosis (FSGS) is a form of glomerulonephritis that develops in various kidney lesions. Because a renal biopsy is not routinely performed especially in proteinuria in the nephrotic range, rigorous estimation of the incidence of FSGS in children is hindered [22]. In our case, the child presented with nephritic-range proteinuria and severe renal failure, so we performed a renal biopsy that revealed chronic glomerulonephritis with segmental and diffuse glomerular hyalinization (sclerosis). Only after the biopsy of the lymph nodes did I find the final diagnosis.

Even though some clinical signs in Fabry disease might appear as clear indicators for diagnosis, delays in recognizing this condition are unfortunately very common, with many years passing between early symptoms and the actual diagnosis [23]. This is due to the wide variety of symptoms that overlap with many other diseases, but also to a poor acknowledgment of this pathology as differential diagnosis in varying clinical setups. This leads to underdiagnosed children, that will finally be correctly identified as Fabry patients well into adulthood. Therefore, there is a great need of raising awareness of this fact, as ERT—if initiated as soon as possible—can positively impact these patient’s outcomes to a degree where kidney transplantation for example, as in our clinical case, might be avoided.

Another way to overcome the issue of underdiagnosis is to implement newborn screening programs that can identify patients before developing symptoms. Newborn screening for Fabry disease, the best way to detect the disease early, before the onset of symptoms, is currently being implemented in Taiwan and several states in the United States of America [24]. However, these are not available world-wide and their sensibility and specificity must also be taken with caution so clinical suspicion is of the essence and must be cultivated.

Diagnosis of Fabry disease can be established by genetic testing (GLA gene located on Xq22.1), measurement of α-Gal A enzyme activity or analysis of either Globotriaosylceramide (Gb3) in peripheral blood mononuclear cells or Globotriaosylsphingosine (LysoGb3). Various mutations, including missense/nonsense mutations, splice defects, regulatory abnormalities, small deletions and insertions, small indels, gross deletions and insertions, and complex rearrangements associated with the GLA gene, have been discovered over the last 30 years [25]. Pathogenic mutations of the GLA gene can lead to a decrease to the disappearance of enzyme activity by affecting the synthesis, processing and stability of α-Gal A or by modifying the hydrophobic core of the protein [26,27]. Certain mutations of GLA gene (OMIM 300644) cause complete loss of function, being linked to classical form of Fabry disease with severe phenotypes, while other mutations might cause late-onset disease with milder clinical manifestations. These can be searched either by genotyping or trough sequencing analysis. The latter might reveal variants of unknown significance which must always be well documented and analyzed in relation to a-Gal α-Gal enzyme activity and clinical manifestations, to determine their pathogenicity.

α-Gal A enzyme activity measured in dried blood spots, serum or plasma is useful in evaluating males, but not reliable for detecting manifesting heterozygous females, as in their case an only slightly decreased α-Gal A or even normal activity might be present. For this situation, measurement of LysoGb3—a degradation product of Gb3—might be more efficient while also representing an indicator of disease activity, along with Gb3 detection in peripheral blood mononuclear cells [23].

Considering the renal involvement in Fabry disease, apart from classical investigations such as albuminuria, proteinuria, β2-microglobulin, urinary microscopy, creatinine, serum urea, uric acid, GFR, ultrasound and kidney biopsy, one should also take in consideration potential new biomarkers such as urinary Gb3, uromodulin, prostaglandin H2 D-Isomerase and bikunin [28].

Regarding treatment in Fabry disease, there are two kinds of therapies: enzyme replacement therapy (agalsidase alfa; agalsidase β) and chaperone therapy (migalastat).

Substitution trough ERT has been shown to decrease Gb3 accumulation in liver and in tubular epithelial cells, with a consequent reduction of Gb3 excretion in urine and reduction in podocytes Gb3 inclusions [28]. Moreover, a decrease in glomerular hyperfiltration was also linked to ERT. The initiation of ERT therapy as early as possible gives the best clinical outcome, but the effect depends on the stage of the disease [29]. ERT should be considered in cases of Fabry disease of both sexes, symptomatic cases, in acute cases where neuropathic pain predominates, pathological albuminuria (≥3 mg/mmol creatinine), severe gastrointestinal symptoms or cardiac involvement [18,30]. Unfortunately, in the case of our patient, the referral was late, after the onset of renal failure, with the appearance of hypertensive complications and the development of cardiopathy secondary to the disease. Recent studies reveal that ERT can reduce Globotriaosylceramide deposits in the kidney, heart and skin, being particularly effective in endothelial cell clearance. On the other hand, podocytes, distal tubular cells, and smooth muscle cells showed less reduction in Globotriaosylceramide than that observed in other cell types, therefore, it appears to be more resistant to ERT [31]. The clinical benefit of ERT is mainly observed in patients who initiate ERT before the presence of irreversible organ damage, as happened in the case of the patient presented [32]. He was presented to our service when the kidney damage was irreversible, and kidney transplantation was the only curative option in his case. Arends et al. observed that, despite treatment with ERT, disease progression is predicted by the presence of renal failure and proteinuria at the time of initiation of therapy [33]. Unfortunately, a downside of ERT is the development of Immunoglobulin G antibodies that might limit its positive effects [26]. Immunoglobulin G antibodies may be generated in about 40% of Fabry males with no α-Gal A activity in response to ERT, and lead to inhibition of enzyme activity that may negatively influence the clinical outcome of Fabry patients [34].

Chaperone therapy with Migalastat stabilizes α-Gal A mutated protein, protecting it from degradation in the lysosomes. However, this kind of treatment can only be used in certain patients showing specific mutations [35]. Migalastat (Galafold, Amicus Therapeutics), was approved in 2016 in Europe and 2018 in the USA, respectively. Chaperone therapy may be used in patients with missense mutations, with reduced catalytic activity [36]. The patient of the study presents a non-amenable mutation, so is not indicative of this therapy for him. Moreover, Migalastat is significantly eliminated by the kidneys and is not recommended in Fabry cases with GFR < 30 mL/min/1.73 m^2^ or ESRD that requires dialysis, as in in our case [37]. In the case of GFR >30 mL/min/1.73 m^2^ is not necessary the dose adjustment [38]. Based on the review of Weidemann, it is important to classify the GAL gene mutation for amenability to treatment with Migalastat in each new Fabry case [35,36]. In this moment Migalastat is the only alternative to ERT in cases where ERT response is lost or in cases of antibody formation [39].

Additional therapy is substrate reduction therapy that targets glycosphingolipid synthesis to reduce the formation of metabolites that cannot be degraded. At this time, it is only available in clinical trials and is not authorized to treat patients [28,34,40].

For Fabry disease patients with amenable mutations, chaperone treatment is the appropriate approach, while for the rest of Fabry disease patients, combination therapy, such as ERT with substrate reduction therapy, might have a beneficial effect [28].

The management of these cases must include a multidisciplinary teamwork, which includes the geneticist, nephrologist, cardiologist, neurologist, gastroenterologist and ophthalmologist. In the present case, one of the problems of therapeutic conduct following the diagnosis was the kidney damage. Although cardiac and neurological damage improved significantly following substitution therapy and while renal formations disappeared, kidney function did not resume, the patient subsequently needing a kidney transplant.

## 4. Conclusions

We presented the rare case of an 11-year-old male patient who came to our clinic in end stage renal failure. We found out that he had palmo-plantar pain for three years, without any identifiable cause until that moment. During the investigations, the presence of multiple organ dysfunction was proven: neurological, cardiac, renal and ophthalmological damage, in the context of which the dosage for multiple storage diseases was reached and the late diagnosis of Fabry’s disease was established. This 3-year delay led to the total compromise of renal function.

## Figures and Tables

**Table 2:** Serological tests.

Investigation	Result
Ag HBs	▪ negative
Ac anti VHC	▪ negative
Ac anti HIV	▪ negative
ASLO	▪ negative
Rheumatoid factor	▪ negative
ANA 9	▪ negative
Ac p—ANCA	▪ negative
Ac c—ANCA	▪ negative
Cryoglobulines	▪ negative

**Table 3:** Other investigations.

Investigation	Result
Lymph node biopsy	▪ complete deletion of the normal architecture of the ganglion (Figure 11) ▪ nests of lymphocytes and remnants of lymphoid follicles ▪ areas of macrophages with foamy cytoplasm (Figure 12) ▪ beaches of macrophages with eosinophilic, homogeneous cytoplasm
Ophthalmological examination	▪ “Swirling” subepithelial opacities in the lower half of the cornea ▪ No tortuosity or vascular dilatations on the retina or conjunctiva ▪ Visual acuity was normal in both eyes ▪ No signs of hypertensive retinopathy
